# The Influence of Variable Stiffness of the Shape Memory Alloys Carbon Composite Structure on Mechanical Vibration

**DOI:** 10.3390/ma17020480

**Published:** 2024-01-19

**Authors:** Damian Markuszewski, Mariusz Wądołowski, Arkadiusz Krajewski

**Affiliations:** 1 Faculty of Automotive and Construction Machinery Engineering, Warsaw University of Technology, 02-524 Warsaw, Poland; mariusz.wadolowski@pw.edu.pl; 2 Faculty of Mechanical and Industrial Engineering, Warsaw University of Technology, 02-524 Warsaw, Poland; arkadiusz.krajewski@pw.edu.pl

**Keywords:** SMA, smart structures, composite materials, mechanical vibration

## Abstract

The purpose of this study is to investigate the dynamic properties of new structures formed by combining carbon fiber and epoxy resin-based composite materials with SMA (shape memory alloy) “smart materials” in the form of NiTiNol wire. Such a combination will have an impact on the dynamics of the structure, especially in terms of stiffness controllability. Key mechanical parameters such as natural frequency and stiffness, as well as the effect of temperature, were determined through experimental studies.

## 1. Introduction

In recent years, there has been an extraordinarily rapid development in engineering materials. Modern engineers now have access to a range of entirely new plastics, whose capabilities are not only constantly evolving but often surpass existing technological capabilities.

A prime example of this advancement is seen in composite materials, which demonstrate significant innovative potential across various technological sectors. Notably, these include aviation [1,2,3], the automotive industry [4], and space technology [5,6], among others. The primary benefits of composites lie in their excellent strength-to-weight ratio, high stiffness, and versatility in shaping. The objective of our research is to explore the dynamic properties of novel structures created by merging carbon fiber and epoxy resin-based composites with “smart materials” such as SMA (NiTiNol). By integrating materials with differing dynamic characteristics, we have developed a structure with entirely new properties. This fusion allows for the utilization of both the benefits of composite materials and the unique features of “smart materials”, namely the ability to modulate the structure’s dynamics through stiffness control. The classification of fiber-reinforced composites is typically based on the matrix type utilized. To date, the most prevalently used composites in technological applications are those reinforced with glass, carbon, and aramid fibers (fabrics).

**Figure 1:**
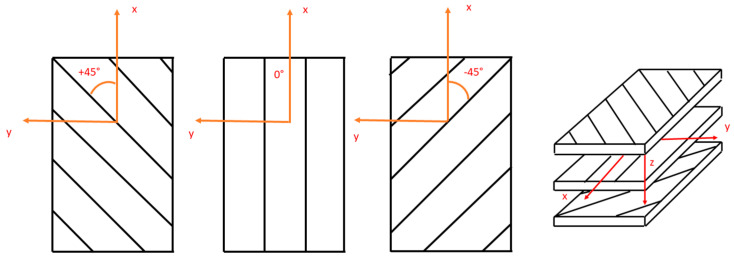
Example of structure and angular orientation of laminate layers [7].

The research should begin with designing and fabricating a series of specimens with varying properties, initially excluding SMA (Shape Memory Alloys). These composite structures will differ based on the type of material used in each layer, the number of layers, and the angular orientation of the fibers in the matrix (Figure 1).

The experiments conducted will aim to determine the basic mechanical properties [8,9,10] such as Young’s modulus and density, as well as the impact of temperature on these properties. In parallel with the design and fabrication of the specimens, a test rig for evaluating the new structures was developed. A fixture for testing thin-walled structures both statically and dynamically was also constructed.

Given the specific properties critical in certain structural applications, the use of materials known as “smart materials” is increasingly prevalent. The concept for researching a new group of smart materials emerged from the need to influence the stiffness of structures, particularly the ability to control certain parameters affecting the vibration patterns of mechanically thin-walled structures. Such effects can be achieved using materials capable of reversible changes in shape, size, or density, including self-assembly capabilities.

The primary challenge in creating such a material lies in combining a carbon composite, an effective electrical conductor, with a shape memory alloy (SMA) wire, without exceeding the glass transition temperature of the resin. The SMA’s heating is designed to facilitate a reversible transformation, allowing it to return to a predefined shape under suitable conditions. Therefore, appropriate thermal and electrical insulation is necessary. The SMA wire, serving as a matrix, will not only revert to its original shape through phase transformation but also enable control over the structure’s stiffness and vibration characteristics. Additionally, the integration of a smart material within the composite structure can function as a mechanical energy absorber, mitigating stress and enhancing resistance to fracture. The bidirectional shape memory effect is particularly desirable in this context, where the smart material ‘remembers’ the shapes associated with its high-temperature or low-temperature phase, with the transition between these shapes triggered by temperature changes [11].

**Figure 2:**
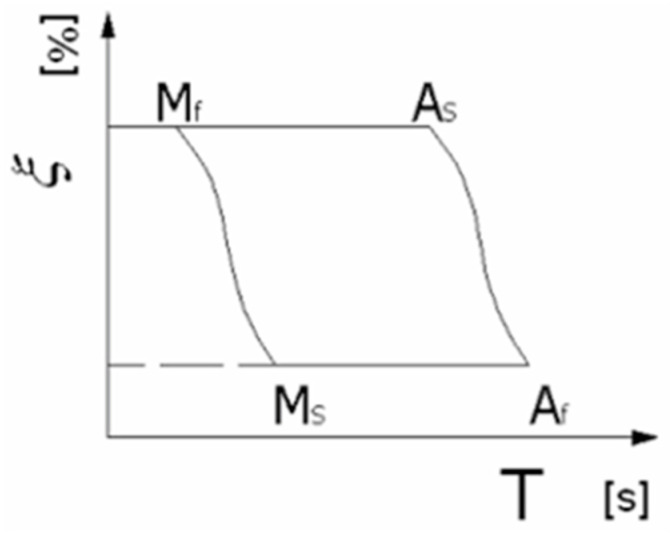
Graphical model of martensite–austenite transformation [11].

The SMA (Shape Memory Alloy) material itself presents a distinctive structure. It has the ability to change shape upon heating to a specific transformation temperature, which facilitates the phase transition between austenite and martensite. This memory effect can be either unidirectional or bidirectional. The unidirectional effect manifests when martensite is heated to the high-temperature austenite phase, assuming the shape set in this phase. In contrast, the bidirectional effect is a reversible process that occurs without any stress application. Additionally, there is a pseudo-elastic strain effect (Figure 4), which is deformation resulting from stress-induced martensitic transformation. These phase transformations are accompanied by changes in the material’s structure: austenite, a high-temperature phase, has a cubic crystal structure, whereas martensite, a low-temperature phase, features an oblique or twinned structure. These transformations are initiated when specific limiting temperatures, well documented in the literature and depicted in Figure 2 [12], are surpassed:

- The martensitic transformation starting temperature (Ms), where austenite begins its transformation into martensite;
- The martensitic transformation finishing temperature (Mf), where the transformation completes, resulting in the entire material being in the martensite phase;
- The austenitic transformation starting temperature (As), beyond which the reverse transformation (martensite to austenite) commences;
- The austenitic transformation finishing temperature (Af), at which point the entire material is in the austenite phase.

The phenomenon of phase transformation under load application is well established. This effect involves applying a load to a material in its low-temperature phase, which has a twin martensite structure, causing a transition to a distorted twin martensite structure. When the load is removed, the material does not revert to its original phase; this reversal is only possible after heating and transitioning through the austenite phase. Conversely, applying a load to the material in the austenite phase results in a phase transformation to a distorted twinned martensite. It is important to note that the greater the applied load, the higher the transformation temperatures will be. Another noteworthy effect is pseudoelasticity, characterized as a phase transformation that occurs when a load is applied without a change in temperature. This transformation results in a near-twisted distorted martensite, accompanied by very high strain.

**Figure 3:**
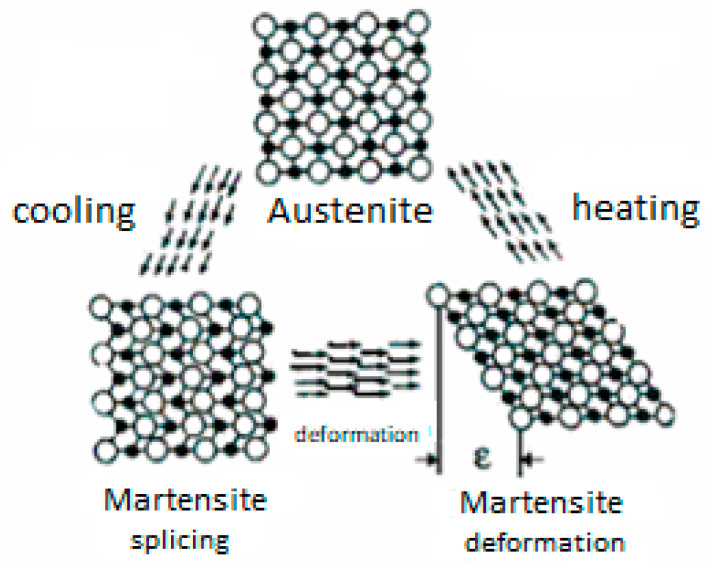
Crystal structure transformation—Nitinol [11].

In modern technology, the most commonly used shape memory materials are various titanium–nickel alloys, collectively referred to as Nitinol (Figure 3). The transformation temperature of Nitinol is influenced by its chemical composition and spans a wide range, from −50 °C to +110 °C. The transformation temperature can be reduced by adding alloying elements such as Fe, Cr, Co, and Al, while it can be increased by adding elements like Hf, Zr, Pd, Pt, or Au. The width of the hysteresis loop can be modified by adding Cu—Table 1 (which decreases it), or Nb (which increases it). To enhance the wire’s strength, elements such as Mo, W, O, and C are added [13]. Nitinol wires are produced through vacuum melting of metals at temperatures exceeding 1250 °C. The density of this alloy is 6.5 g/cm^3^. Among SMA materials, Nitinol exhibits superior properties: it can undergo reversible deformation of up to 8%, and the stress during recovery can reach 800 MPa [13].

The endeavor to integrate carbon components with SMA wires into a single composite could, according to the authors, mark a substantial advancement in the field of smart materials. This integration aims to create a structure that combines all the positive attributes of composites (like carbon, aramid, glass) with the ability to alter its dynamic characteristics during observation. This unique feature potentially introduces extensive applications in various fields.

SMA alloys are characterized by varying transformation temperature ranges and the size of their hysteresis loops. Additionally, the equivalent Young’s modulus (used for computational purposes) varies depending on the strain rate. It is important to note that the material properties are sensitive to the angular orientation of the layers and that the elastic characteristics exhibit nonlinearity. In composite structures [19], analytical calculations often yield results that significantly differ from experimental findings. This discrepancy arises because the material characteristics of the composite only become fully apparent after the product’s formation, necessitating empirical studies on the final product.

Adaptive structures have become an increasingly prominent research area, with a notable surge in publications and patents since 2000 [20]. Their wide range of properties and mechanisms, leading to diverse potential applications, offer significant potential for innovation in various technological fields. These include aerospace [21,22,23,24], medicine [25,26,27], automotive [28], and robotics [29,30,31], among others.

## 2. The Model

The aim of the model is to determine the equivalent Young’s modulus and equivalent stiffness of the composite structure formed by combining carbon fiber and SMA wire infiltrated with epoxy resin. For this purpose, the authors chose an indirect method by measuring the natural frequency of the vibrating beam and using a well-known model for a flexural beam.

**Figure 4:**
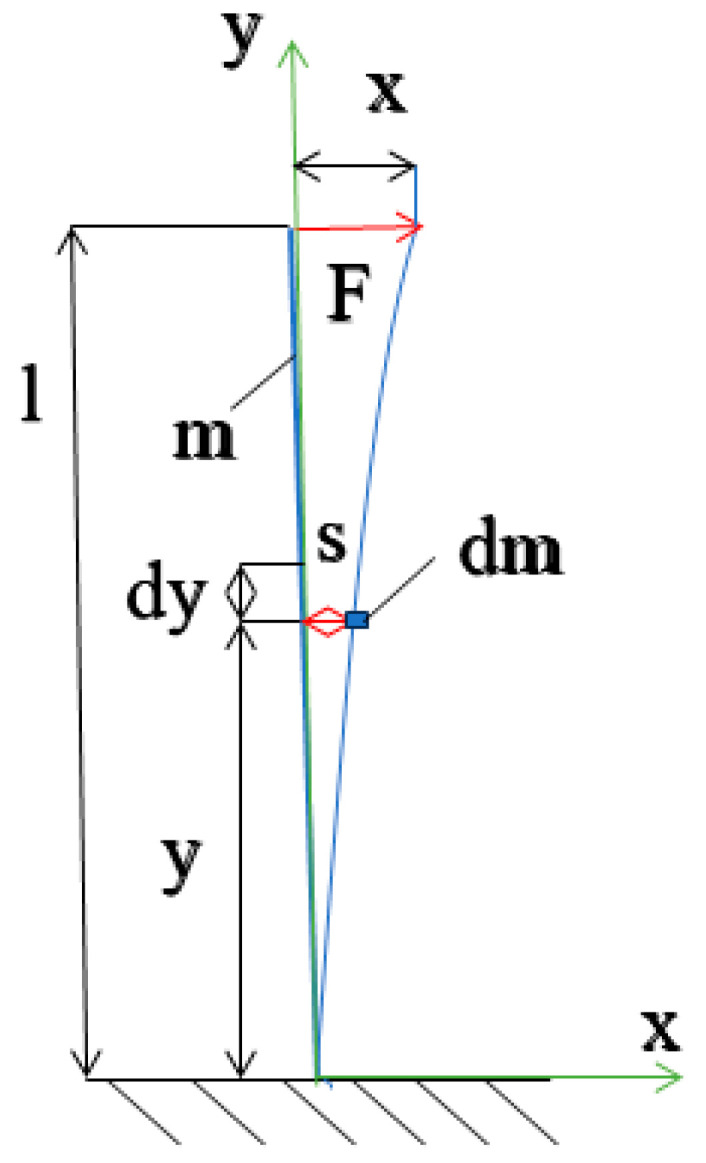
A model of an oscillating beam.

As a reference model, consider an oscillating system with one degree of freedom, represented as a vertically positioned beam of length *l* and mass *m*, with its lower end restrained (Figure 4). This is a resilient and flexible composite beam with a constant cross-section, fixed at one end, similar to a cantilever beam as depicted in Figure 1. The free end of the beam is displaced from its equilibrium position by a static force *F* along a path *x.* The fundamental relationships governing the oscillatory motion of such a system can be described by the following Equation (16):

Approximate methods are often employed to determine the natural frequency of such a system, and in this case, the Rayleigh method is utilized. The Rayleigh method is an energy-based approach that leverages the principle of conservation of energy in a system moving within a potential force field [32]. This necessitates the calculation of the maximum potential energy (occurring at maximum deflection) and the maximum kinetic energy (at the point of maximum velocity). To achieve this, it is essential to establish a description of the deformation curve of the beam during oscillation. Consequently, the kinetic energy (1) associated with the movement of this element can be expressed as follows:

(1) $$E_{k}=\frac{1}{2}\int_{0}^{l}dm{\dot{s}}^{2}$$

where *dm* represents the mass of a three-dimensional beam element with a cross-section *a* = *b* × *h* and length *dy*. Here, *b* denotes the width of the composite beam, while *h* indicates the height of the beam in the direction of displacement and bending. The term *q* refers to the specific density of the composite beam. Consequently, the mass of the element can be expressed as follows:

(2) dm=aqdy

We will determine the value of the displacement of a point at distance *y* using the equation of the beam deflection line:

(3) $$s=x\frac{y^{2}\left(3l-y\right)}{2l^{3}}$$

Suppose that a beam element dm away from the attachment point at a distance *y* moves with velocity:

(4) $$\dot{s}=\dot{x}\frac{y^{2}\left(3l-y\right)}{2l^{3}}$$

The energy of the element *dm* of the beam is:

(5) $$E_{k}=\frac{1}{2}\int_{0}^{l}aq{\dot{s}}^{2}dy$$

(6) $$E_{k}=\frac{1}{2}aq{\dot{x}}^{2}\int_{0}^{l}\frac{y^{4}{\left(3l-y\right)}^{2}}{4l^{6}}dy$$

(7) $$\int_{0}^{l}\frac{y^{4}{\left(3l-y\right)}^{2}}{4l^{6}}dy=\frac{1}{4l^{6}}\int_{0}^{l}(9l^{2}y^{4}-6ly^{5}+y^{6})dy=\frac{1}{4l^{6}}\left(\frac{9}{5}l^{2}y^{5}-ly^{6}+\frac{1}{7}y^{7}\right){|}_{0}^{l}=\frac{33}{140}l$$

(8) $$E_{k}=\frac{1}{2}\frac{33}{140}{aql\dot{x}}^{2}=\frac{1}{2}\frac{33}{140}{m\dot{x}}^{2}$$

If tilted out of equilibrium by *x*, the deformed beam will have potential energy in the form of elastic energy:

(9) $$W=\frac{1}{2}Fx$$

where *W*—the work put in by applying a static force *F* will cause a pivot of *x* value:

(10) $$x=\frac{FL^{3}}{3EI}$$

where *x*—deflection arrow of the composite beam, *E*—equivalent Young’s modulus of the composite beam, *I*—moment of inertia of the composite beam section.

While the value of the force will be:

(11) F=kx

where *k*—the elastic coefficient of the bent composite beam.

(12) $$k=\frac{3EI}{L^{3}}$$

The elastic energy will be determined by the formula:

(13) $$E_{p}=\frac{1}{2}kx^{2}$$

After the final substitution:

(14) $$E_{p}=\frac{3EI}{2L^{3}}x^{2}$$

The total energy of this system, will be:

(15) $$E_{c}=\int_{0}^{l}\frac{1}{2}aqdy{\left(\frac{\partial s}{\partial t}\right)}^{2}+\frac{3EI}{2L^{3}}x^{2}=const$$

The equation of motion is a multidimensional generalization of the well-known equation describing the vibration of a system with one degree of freedom. In practice, we often consider various special cases of vibration, for which the equation takes simpler forms. If we assume that the force vector *F* is equal to zero, then we are dealing with the so-called free vibration—without external loads. If, in addition, there is no damping in the structure, then the vibration is called free vibration and the differential equation of the deflection line of the bent beam has the form:

(16) $$\left(\frac{33}{140}aql\right)\frac{d^{2}x}{dt^{2}}+\frac{3EI}{l^{3}}x=0$$

On the other hand, the general solution of the differential equation:

(17) $$x=Asin\left(\omega_{0}t+\varphi \right)$$

(18) $$\dot{x}=A\omega_{0}\cos\left(\omega_{0}t+\varphi \right)$$

Substituting:

(19) $$E_{k}=\frac{1}{2}\frac{33}{140}aql\;A^{2}\omega_{0}^{2}{cos}^{2}\left(\omega_{0}t+\varphi \right)$$

(20) $$E_{p}=\frac{3EI}{2L^{3}}A^{2}{sin}^{2}\left(\omega_{0}t+\varphi \right)$$

From the principle of conservation of energy: *E_k_ max = E_p_ max*

(21) $$\omega_{0}=\sqrt{\frac{k}{m}=}\sqrt{\frac{3EI}{\left(\frac{33}{140}aql\right)l^{3}}}$$

## 3. Experiment

The subject of the analysis is the observation of vibrations at the free end of a composite beam with an SMA wire loop core, caused by tilting out of the equilibrium position through static forces F and its subsequent removal. The deflection line shape corresponding to the first vibration mode of the SMA composite beam is shown in Figure 1. It resembles the deflection line of a beam loaded with a concentrated force at the free end. The system’s vibration frequency corresponding to the first vibration mode is termed the fundamental frequency. If the analysis is limited to vibrations at the first natural frequency, such a system can be approximated, with some limitations, as a one-degree-of-freedom system, where:

(22) $$\omega_{0}=2\pi f$$

(23) $$f=\frac{\omega_{0}}{2\pi }$$

(24) $$\omega_{0}=\sqrt{\frac{3EI}{\left(\frac{33}{140}aql\right)l^{3}}}$$

(25) $$f=\frac{1}{2\pi }\sqrt{\frac{3EI}{\left(\frac{33}{140}aql\right)l^{3}}}$$

The tested samples were prepared as composite strips with parameters outlined in Table 4. The study presents results for the five most representative configurations listed in Table 2.

**Table 2:** Structure of the tested samples (Figure 5).

Sample	Structure
Plytka_01-1	three layers of carbon fiber mat, interlaced plain 1/1, weight 200 g/m^2^, weave density 5/5, thickness 0.3 mm, angular orientation of layers 90/0/90, loop of SMA wire outside with glue—diameter ø 0.7 mm—cold
Plytka_02-1	three layers of carbon fiber mat, interlaced plain 1/1, weight 200 g/m^2^, weave density 5/5, thickness 0.3 mm, angle orientation of layers 90/0/90
Plytka_03-1	three layers of carbon fiber mat, interlaced plain 1/1, weight 200 g/m^2^, weave density 5/5, thickness 0.3 mm, angle orientation of layers −45/0/45
Plytka_05-1	three layers of carbon fiber mat with aramid, plain 1/1 interlacing, weight 200 g/m^2^, weave density 5/5, thickness 0.3 mm, angle orientation of layers 90/0/90
Plytka_07-1	three layers of fiberglass mat, weight 200 g/m^2^, thickness 0.3 mm
Plytka_10Z-1	three layers of carbon fiber mat, interlaced plain 1/1, weight 200 g/m^2^, weave density 5/5, thickness 0.3 mm, angular orientation of layers 90/0/90, loop of SMA wire—diameter ø 0.7 mm—cold
Plytka_10G-1	three layers of carbon fiber mat, interlaced plain 1/1, weight 200 g/m^2^, weave density 5/5, thickness 0.3 mm, angular orientation of layers 90/0/90, loop of SMA wire inside—diameter ø 0.7 mm—hot
Plytka_11Z-1	only loop of SMA wire—diameter ø 0.7 mm—cold
Plytka_11G-1	only loop of SMA wire—diameter ø 0.7 mm—hot

Epidian 5 resins, with properties shown in Table 3, were used as the binding element in all samples. The samples were made by hand lamination, removing excess resin and air bubbles with a roller. The mass ratio of resin to reinforcement was 40% to 60%.

Sample Plytka_10Z-1 was prepared as a composite strip, embedding an SMA wire loop (diameter ø 0.7 mm) as additional reinforcement, with connections extending outside the sample (Figure 10). The smart material was electrically insulated from the composite and connected to a current source. Upon activating the current source, the SMA material transformed, altering the system’s parameters. A LW LONGWEI—K3010D pulsed laboratory power supply with a 30 V and 0.1 V resolution, and 10 A with 0.01 A resolution, was used to power the SMA wire loop. The voltage during measurement was 2 V, with a current of 3.5 A (Table 4).

**Figure 6:**
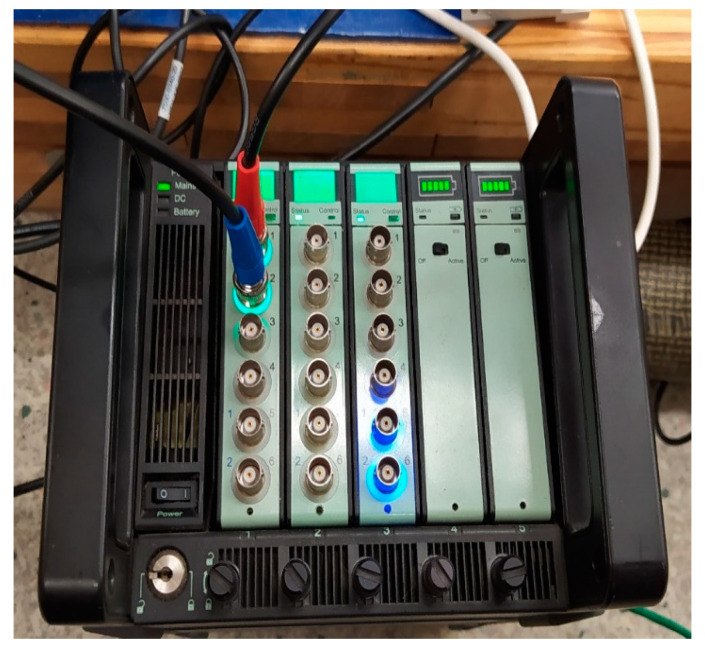
B&K measurement trac.

The test setup included a B&K Type 3660-C docking station with an embedded Type 3160-A-4/2 measurement card (Figure 6).

**Figure 7:**
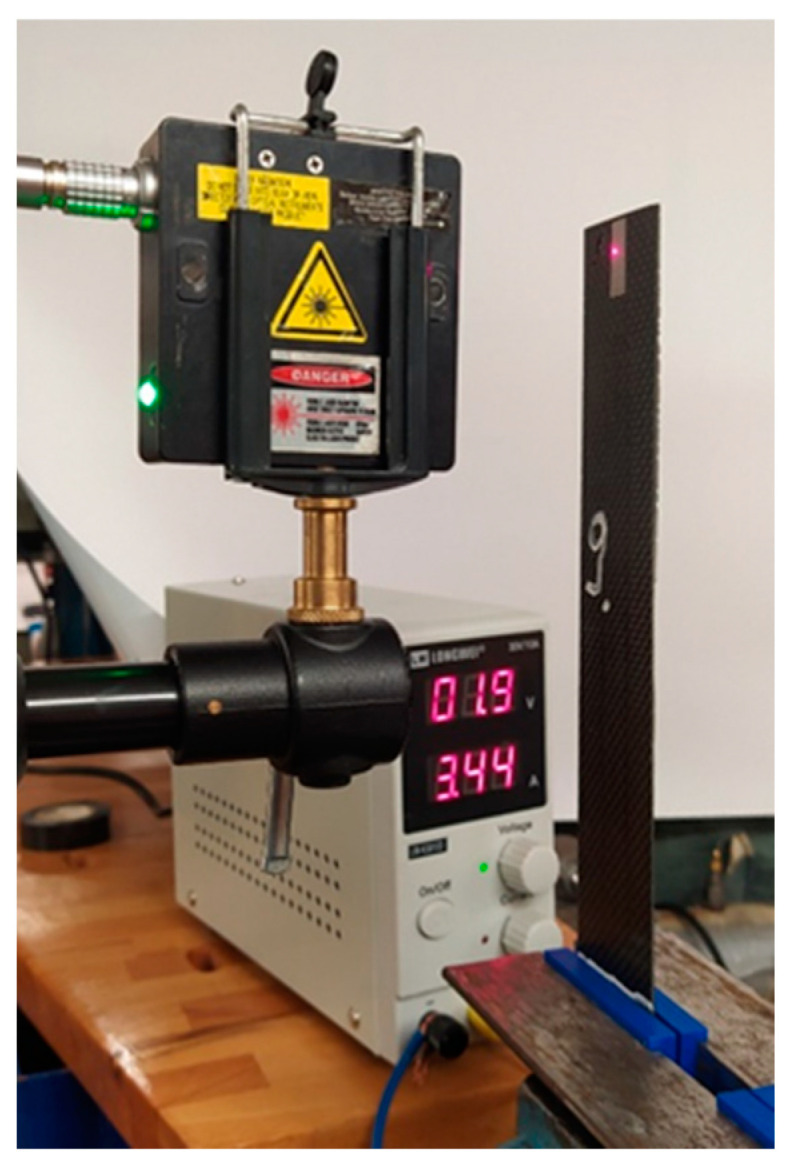
Laser for measuring (vibration displacement).

**Figure 8:**
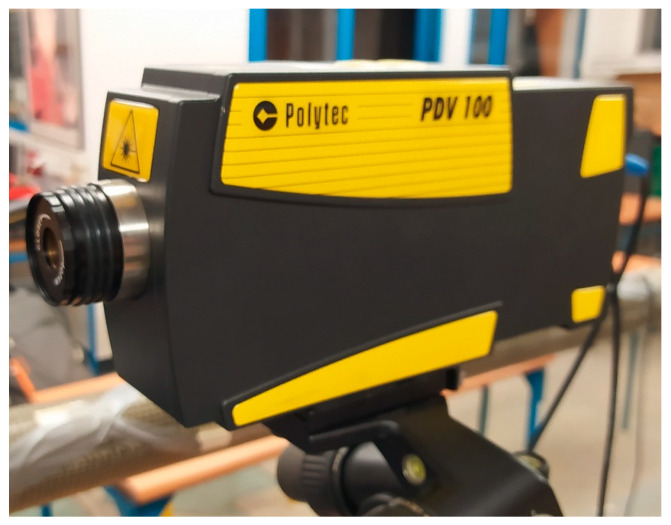
Laser for measuring (vibration speed).

The measurement tracks also included lasers: for displacement measurement KEYENCE LK-G152 (Figure 7) with a measurement range of +/−39 mm for 1000 Hz, and for vibration velocity measurement Polytec Vibrometer PDV 100 (Figure 8) with a measurement range of 0.5–22 kHz (for three ranges of 5/25/125 mm/s/V at peak 20/100/500 mm/s from a distance of 90 mm to 30 m).

**Figure 9:**
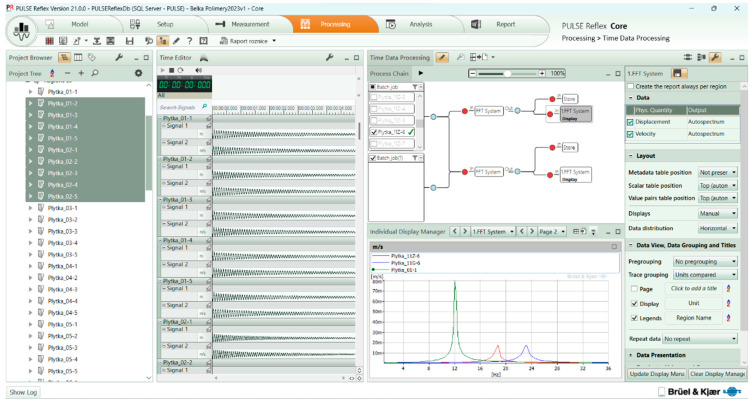
Data acquisition program.

Free vibration fading waveforms were recorded over ten seconds at a sampling frequency of 25 kHz. The data were captured using a PC with PULSE Reflex 21 software (Figure 9), and calculations were performed using MATLAB R2021b software, The MathWorks Inc. (9.11 version, Natick, MA, USA).

## 4. Results and Discussion

Despite the potential to determine frequencies of successive natural vibration modes using computer programs, this study was limited to determining the fundamental frequency only. The equivalent Young’s modulus and stiffness were calculated using transformed equations based on the measured natural frequency [29]. Other dimensions like length, width, and height were measured using calipers. The specific density for both the composite and SMA materials was assumed. Moreover, the moment of inertia relative to the central axis of the section was calculated. The bending vibration model was analyzed according to the elementary theory of bending, assuming that normal stresses in sections parallel to the beam axis equal zero and that flat cross sections of the beam remain flat and perpendicular to the bent axis after deformation.

(26) $$m=m_{b}+m_{w}$$

(27) $$E_{z}=\frac{{\left(2\pi fm\right)}^{2}\left(\frac{33}{140}bhql\right)l^{3}}{3I}$$

(28) $$k_{z}=\frac{3E_{z}I}{L^{3}}$$

where *E_z_*—Young’s equivalent modulus, *k_z_*—substitute stiffness, *f_m_*—natural frequency (measured), *m*—weight of the composite beam with SMA wire.

**Figure 10:**
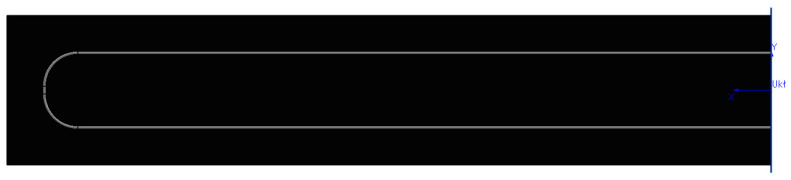
Model of sample Plytka_10Z-1 in SolidWorks 2023.

The practical suitability of the derived formulas and method was verified based on the measured first natural frequency and displacement. Equivalent Young’s modulus and stiffness values were compiled in Table 5. The samples were modeled in SolidWorks 2023 to verify moments of inertia relative to the initial coordinate system. The specific density of the three-layer carbon fiber sample according to the program should be 1780 kg/m^3^, while the density determined from mass and calculated volume is 1.51 g/cm^3^. The density of the SMA wire according to the literature is 6500 kg/m^3^, while from the determined mass and calculated volume it is 6570 kg/m^3^. The wire was modeled as a cylinder placed on the specimen, without structural attachment (Figure 10).

Dynamic measurements involved swinging the free end by 20 mm and observing the quenching vibration upon removing the static force. One end was fixed in a special plastic holder to isolate the system tested from external interference, to bring out the power connectors for sample Plytka_10Z-1. A reflective tape of 0.16 g—omitted during modeling—was applied to the free end of the samples. The actual length of the specimen (240 mm) was reduced by 35 mm to map the attachment.

**Figure 11:**
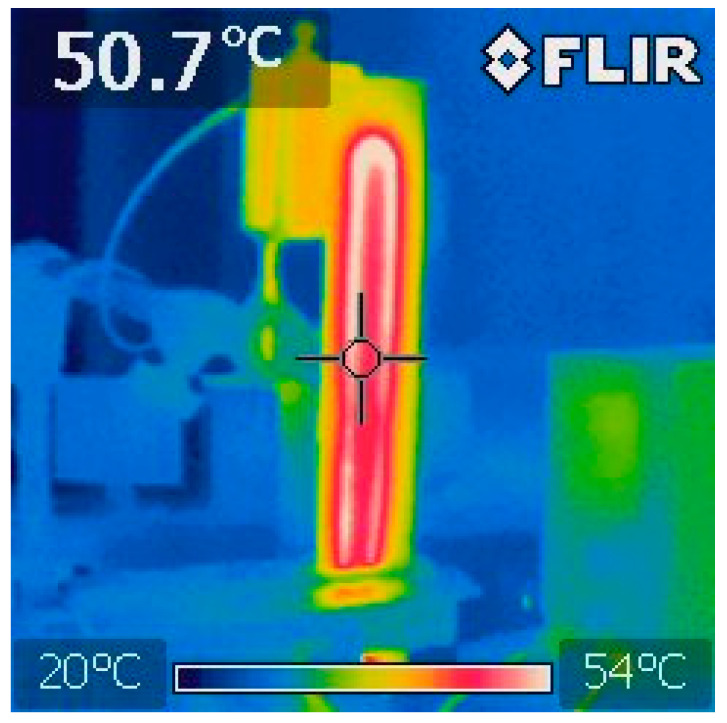
Thermal imaging photo of sample Plytka_10G-1.

**Figure 12:**
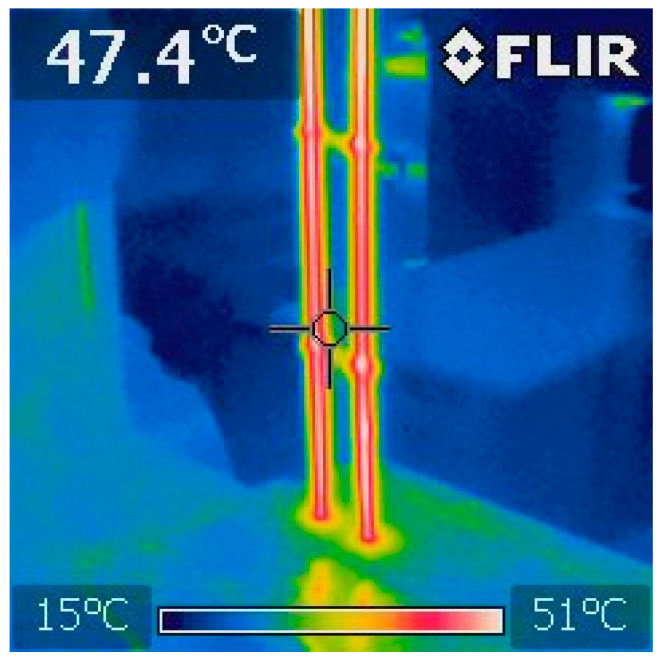
Thermal imaging photo of SMA wire.

Non-contact temperature measurements were also conducted using a FLIR i7 thermal imaging camera. The camera’s specifications are: operating frequency 9 Hz, thermal sensitivity (NETD) 0.1 °C, spectral response 7.5–13 µm, measuring range 20 °C to +250 °C, accuracy ±2% or 2 °C. The transformation of the SMA wire occurred above 50 °C (Figure 11 and Figure 12). The short-term increase in temperature of the composite material, especially Epidian 5 epoxy resin, did not cause damage to the structure or loss of stiffness. Due to the copper alloying additive (NiTiCu—Ni 55.39%, Ti 38.15%, Cu 6.35%), the test wire has a narrower hysteresis compared to binary nickel–titanium wire.

Activating the transformation in the SMA wire, according to the literature, can result in a deformation of 8%; in this case it resulted in a change of 27% in the natural frequency of the composite hot-wire structure, i.e., a shift of the amplitude peak on the spectrum by 5 Hz. This is a large change and translated into a significant increase in the Young’s equivalent modulus value for the new carbon-SMA hot-wire structure and, further, in the equivalent stiffness.

As can be observed, there was an increase in the stiffness of the sample as a result of the addition of the SMA wire compared to the sample with carbon fiber alone, more than doubling. This may be due to a technological phenomenon—the carbon mat does not lay perfectly around the wire, and the free spaces may have been filled with excess resin, which the hand lamination process failed to remove. This can be seen well from the 33% weight increase in the sample with SMA relative to the sample with carbon fiber alone, Where the wire alone is 8.6% of the sample weight; for the hot SMA wire there is a 60% increase in stiffness.

**Figure 13:**
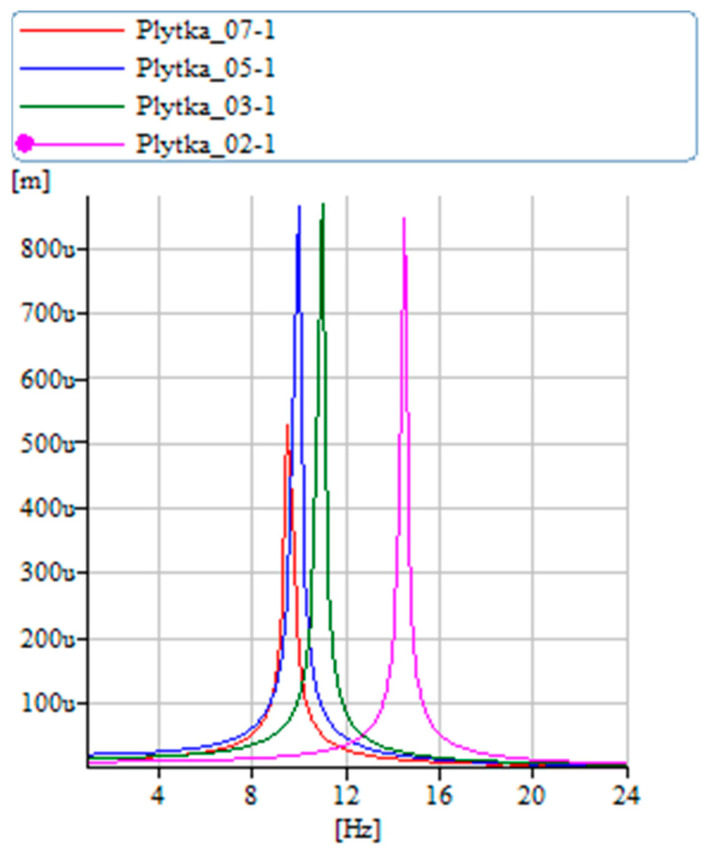
Vibration displacement diagram—samples of different materials.

**Figure 14:**
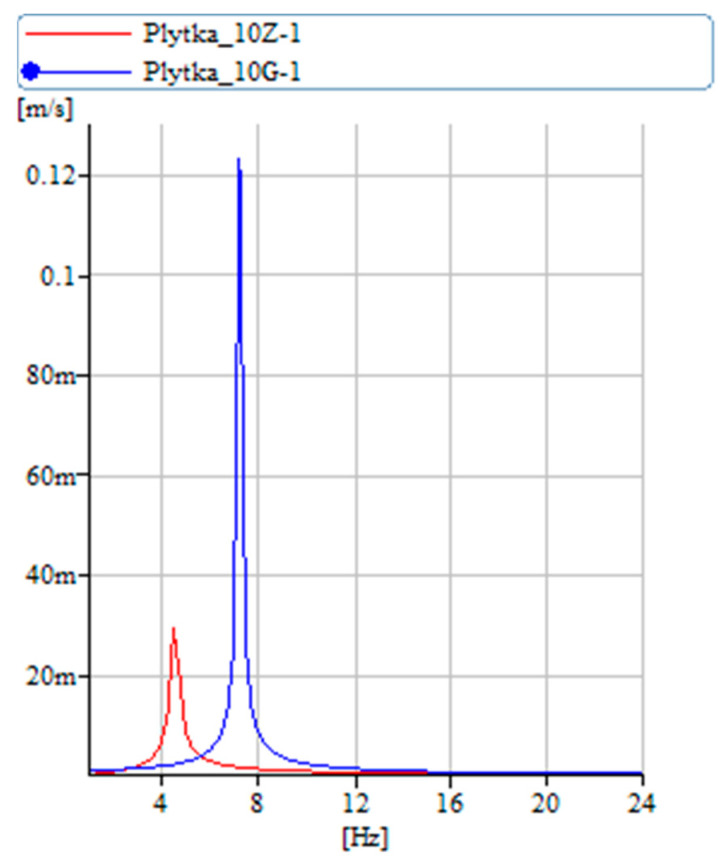
Vibration velocity diagram for only the SMA wire.

**Figure 15:**
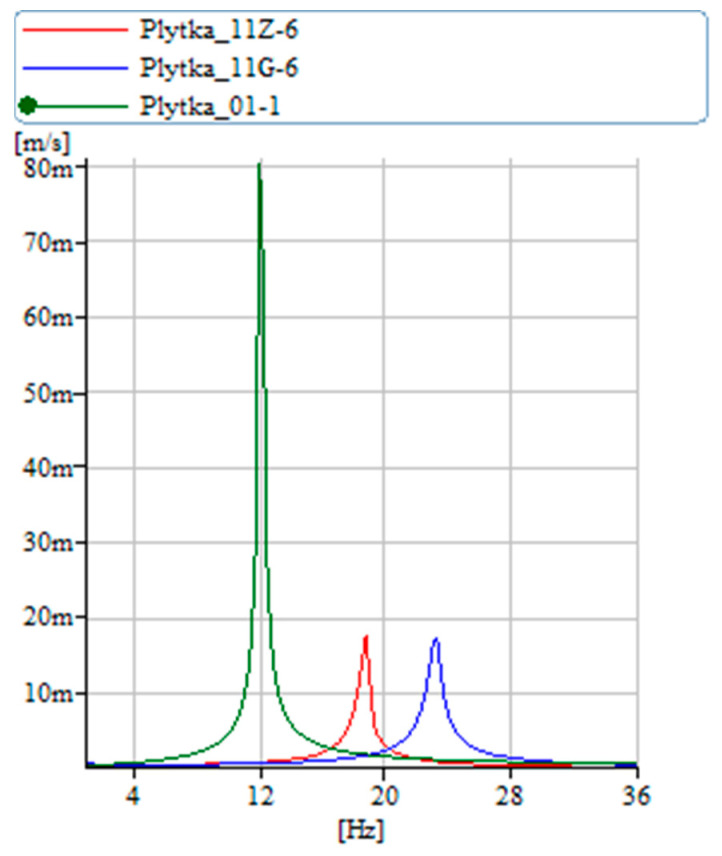
Vibration velocity diagram carbon mat samples, reinforced with SMA wire (wire in 2 states).

**Figure 16:**
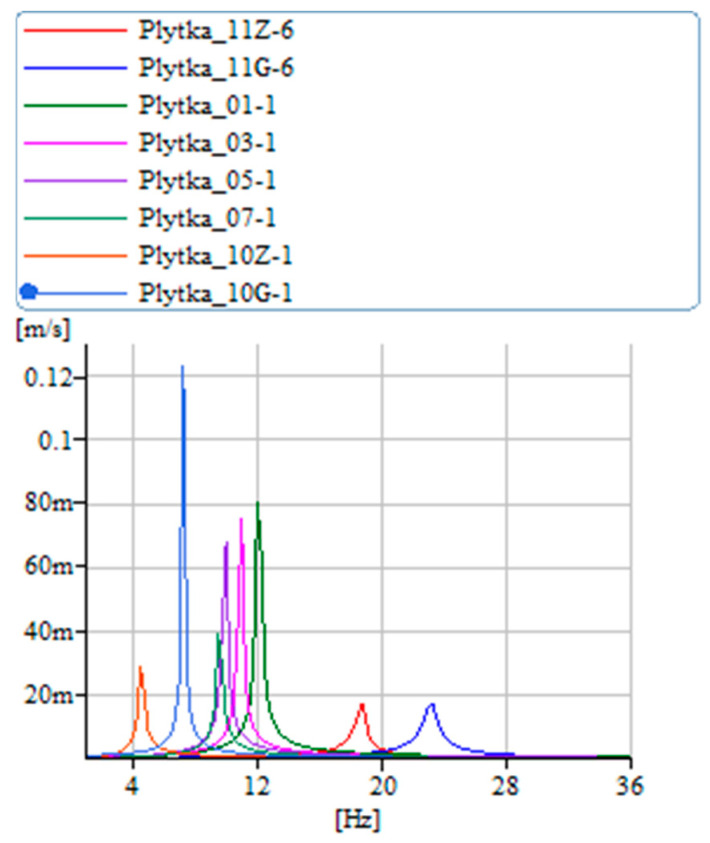
Vibration velocity diagram—carbon mat—tested samples.

Figure 13, Figure 14, Figure 15 and Figure 16 display the amplitudes of vibration displacements at the free end of the restrained composite specimen across various structures. Figure 13 reveals that the fiberglass sample exhibits the lowest amplitude and frequency. This outcome may be attributed to the chaotic arrangement of the glass fibers, impacting the internal friction, and leading to high resin absorption, which in turn affects the specimen’s weight. A more orderly fiber structure, as seen in the carbon samples, likely results in lower internal friction, as indicated by their similar amplitude values. Notably, the carbon sample reinforced with aramid demonstrates greater stiffness compared to those without aramid. The synthetic polymer thread used in this case is poly(p-phenyleneanthylene) (PPTA), also known as para-aramid. Para-aramids are recognized for their exceptional tensile strength and heat resistance, contributing to the increased stiffness observed. Additionally, it is evident that the sample with a +45/0/−45 weave exhibits higher stiffness than the sample with a 90/0/90 weave. This is due to the more active engagement of fibers during bending in the +45/0/−45 weave, as opposed to the 90/0/90 weave where half of the fibers are oriented transversely to the load.

Figure 14 illustrates the vibration frequencies of the SMA wire in isolation. In its unpowered or ‘cold wire’ state, the wire exhibits lower damping and stiffness. Upon activation of the power, transitioning it to the ‘hot wire’ state, the wire undergoes a transformation. This change results in a significant increase in vibration amplitude, approximately fourfold, and an almost 30% increase in stiffness. Figure 15 presents the vibration velocity values for both the pure carbon structure and the carbon structure integrated with SMA, comparing their performance in both ‘cold wire’ and ‘hot wire’ states. It is notable that the vibration velocity of the pure carbon structure attains higher values. Introducing a wire with SMA into the sample stiffens the sample and changes the moment of inertia.

Additional evidence supporting the conclusions drawn from the analysis of characteristics in Figure 14 is that the state in which the transformation is activated by current is marked by increased stiffness, leading to a higher amplitude of vibration velocity. Figure 16 displays the results for the other samples. The fiberglass sample exhibits the lowest amplitude, indicating the highest internal damping, which corroborates the findings of Figure 13. Following this, the carbon sample with a more organized structure shows greater stiffness compared to the regular carbon fiber sample, as well as a higher vibration velocity. The combination of carbon fiber and aramid demonstrates even greater stiffness than both the carbon-only and fiberglass samples, which is also reflected in the increased vibration velocity values.

**Figure 17:**
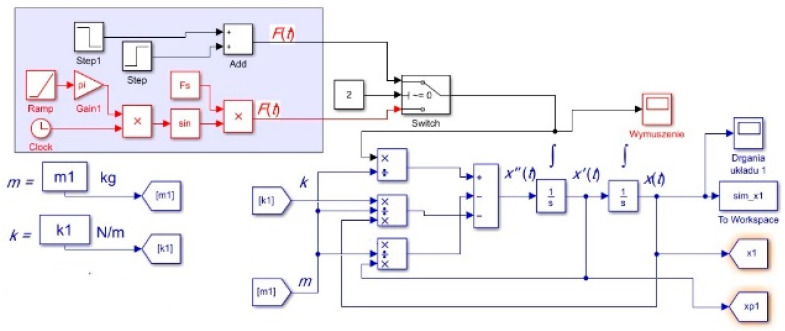
Model in Simulink.

**Figure 18:**
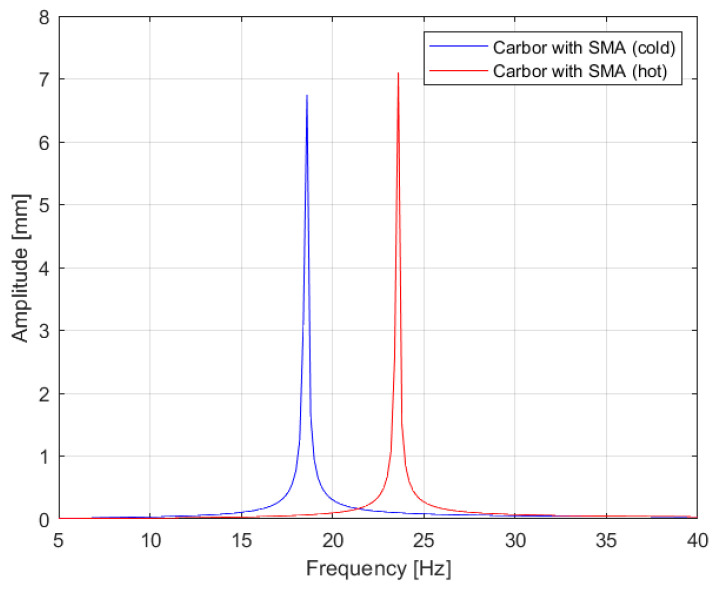
Vibration graph for the obtained results from modeling studies.

A model was constructed using the Simulink software (https://ww2.mathworks.cn/products/simulink.html, accessed on 25 October 2023) (Figure 17), which confirmed the significant impact of the SMA’s transformation on the vibration frequency and stiffness. The results of the analytical calculations align well with both the experimental studies (as shown in Table 5) and the model studies (illustrated in Figure 18), with this alignment further supported by other literature sources [33,34,35]. Heating the wire to its transformation temperature led to a decrease in the natural frequency of vibrations by 5 Hz and an increase in the equivalent stiffness by 60%, which is considered a very favorable outcome.

## 5. Conclusions

The practical significance of analyzing natural vibrations lies in the risk of resonance in elastic structures. Vibration theory teaches that when the frequency of external forces nears the natural frequency of a structure, the amplitude of vibrations increases, potentially leading to resonance. This phenomenon can result in the destruction of the structure. Therefore, the designer’s responsibility is to engineer the elastic or inertial properties of a structure so that its natural frequencies do not align with the frequencies of typical external forces.

The experiment facilitated an understanding of basic mechanical properties (such as equivalent Young’s modulus, density) and how temperature affects these properties. The measure of static stiffness was determined experimentally by evaluating the deflection line within the range of elastic deformation.

SMA-reinforced carbon fiber thermoset composites are noted for their high stiffness and strength. The reinforcing fibers bear tensile loads along their longitudinal axes, while the resin maintains stability among the fibers and absorbs loads perpendicular to the fibers, as seen in compressive or shear loads. Thinner structures are capable of greater deformation.

A key aspect of this approach is using a basic reference model, which demonstrated that results from theoretical models might differ from experimental findings. The integration of carbon components with SMA wires into a single composite, as proposed by the authors, signifies a substantial progression in the field of smart materials. This advancement aims to create structures possessing all the beneficial characteristics of carbon composites, coupled with dynamically changeable properties during observation, thereby broadening the potential applications of these materials.

## Figures and Tables

**Figure 5:**
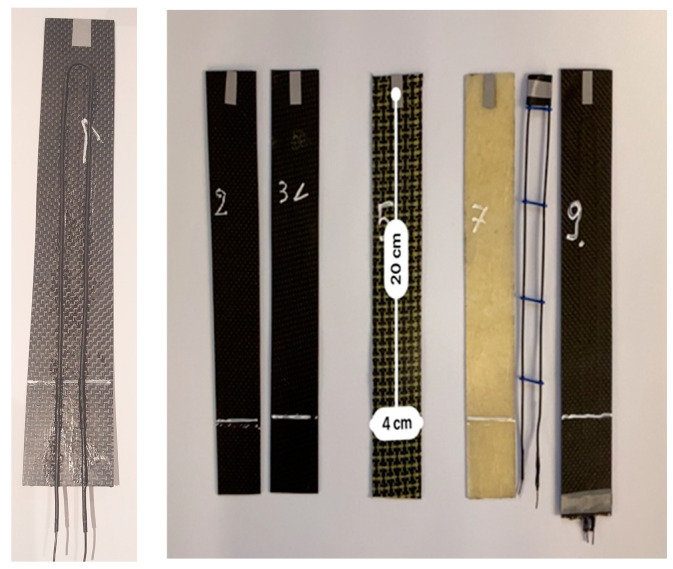
Tested samples (description Table 2).

**Table 1:** Representative transformation temperatures for SMAs with different compositions [14].

NiTi-Based SMAs	Mf	Ms	Af	As	References
Ti50Ni50	15	55	80	89	[15]
Ti38.25Ni55.39Cu6.35	x	45	55	65	tested wire
Ti50Ni40Cu10	21	41	53	67	[16]
Ti40.Ni49.8Hf10	103	128	182	198	[17]
Ti48Ni47Zr5	20	65	75	138	[18]
Ti50Ni45Pt5	10	29	36	49	[15]
Ti50Ni20Pd30	208	241	230	241	[15]

**Table 3:** Properties of Epidian 5 resin.

Sample Number	Structure
density	1.15 g/cm^3^
viscosity	20,000 mPas
gelation time	30 min
curing time	8 h
hardener	Z1—dosage 12 g per 100 g Epidian 5

**Table 4:** Basic data describing the samples.

Fiber Description	Sample	*m_b_* [kg]	*m_w_* [kg]	*h* [m]	*b* [m]	*L* [m]	*V* [m^3^]
Carbon with SMA (hot)	Plytka_10G-1	0.01135	0.00117	0.001	0.040	0.205	0.0000084
Carbon with SMA (cold)	Plytka_10Z-1	0.01135	0.00117	0.001	0.040	0.205	0.0000084
carbon 90/0/90	Plytka_02-1	0.01013	-	0.001	0.040	0.205	0.0000082
carbon +45/0/−45	Plytka_03-1	0.00977	-	0.001	0.040	0.205	0.0000082
carbon with aramid	Plytka_05-1	0.00931	-	0.001	0.040	0.205	0.0000082
glass	Plytka_07-1	0.01109	-	0.001	0.040	0.205	0.0000082

where *m_b_*—weight of the composite beam, *m_w_*—weight of the SMA wire, *h*—height of the sample, *b*—width of the sample, *L*—measurement length of the sample, *V*—volume of the sample.

**Table 5:** Determined quantities describing the phenomenon of vibrations.

Fiber Description	Sample	*q* [kg/m^3^]	*q_SMA_* [kg/m^3^]	*I* [m^4^]	*f_m_* [Hz]	*E_z_* [GPa]	*k_z_* [N/m]
Carbon with SMA (hot)	Plytka_10G-1	1722	6573	3.4 × 10^−13^	23.5	235.53	273.18
Carbon with SMA (cold)	Plytka_10Z-1	1722	6573	3.4 × 10^−13^	18.5	145.97	169.29
carbon 90/0/90	Plytka_02-1	1506	-	3.3 × 10^−13^	14	67.53	78.38
carbon +45/0/−45	Plytka_03-1	1454	-	3.3 × 10^−13^	8	21.26	24.68
carbon with aramid	Plytka_05-1	1384	-	3.3 × 10^−13^	10	31.66	36.75
glass	Plytka_07-1	1723	-	3.3 × 10^−13^	9.5	34.04	39.51

where *q*—specific density of the sample (designated), *q_SMA_*—specific density of the wire (SolidWorks), *I*—moment of inertia (SolidWorks), *f_m_*—natural frequency (measured), *E_z_*—equivalent Young’s modulus of the sample (designated), *k_z_*—equivalent stiffness of the sample (designated).

## Data Availability

Data are contained within the article.
